# Thermosensitive Scattering Hydrogels Based on Triblock Poly-Ethers: A Novel Approach to Solar Radiation Regulation

**DOI:** 10.3390/polym16010008

**Published:** 2023-12-19

**Authors:** Dewei Qian, Siyu Yang, Xiaofang Wang, Yang Tian, Weijia Wen

**Affiliations:** 1 Division of Emerging Interdisciplinary Areas, Academy of Interdisciplinary Studies, The Hong Kong University of Science and Technology, Clear Water Bay, Kowloon, Hong Kong; dqianaa@connect.ust.hk; 2 Thrust of Advanced Materials, The Hong Kong University of Science and Technology (Guangzhou), Nansha, Guangzhou 511400, China; 3 Shenzhen-Hong Kong Collaborative Innovation Research Institute, The Hong Kong University of Science and Technology, Futian, Shenzhen 518000, China; 4 Department of Physics, The Hong Kong University of Science and Technology, Clear Water Bay, Kowloon, Hong Kong; phsidney@ust.hk; 5 Chongqing Hewei Technology Co., Ltd., Chongqing 401120, China; xiaofang.wang@cqhwkj.com (X.W.); tianyang9109@163.com (Y.T.)

**Keywords:** thermosensitive scattering glass, hydrogel, triblock polyether, SHGC, energy-saving

## Abstract

Energy conservation in buildings is paramount, especially considering that glass accounts for 50% of energy consumption. The solar heat gain coefficient (SHGC) of glass is a critical energy-saving index for transparent structures. However, the fixed SHGC of ordinary glass makes it difficult to provide both summer shading and winter heating. In this study, we synthesized a hydrogel with a thermosensitive scattering (TS) property using triblock polyether and acrylamide. This hydrogel can realize the transition of clearness and atomization based on the temperature. When sealed within a glass cavity, it exhibits a high SHGC of 0.682 in its transparent state and a low SHGC of less than 0.31 when atomized. The lower critical solution temperature (LCST) of the TS glass can be adjusted from 0 to 70 °C to suit different regions. The photothermal properties of the material remained stable after 200 hot and cold cycles and 200 h of ultraviolet irradiation. This glass can prevent solar radiation from entering the room in summer, thereby reducing air conditioning usage and power consumption. In winter, it allows solar heat radiation to enter the room, minimizing the need for artificial heating. Its adaptable temperature design makes it an excellent solution for designers to create energy-efficient building exteriors.

## 1. Introduction

Building energy consumption constitutes approximately 40% of the world’s total energy consumption [1,2]. Transparent maintenance structures, predominantly consisting of glass curtain walls and windows, account for half of a building’s total energy consumption [3,4,5]. In an effort to augment the energy-saving efficiency of the curtain wall system and mitigate the building sector’s impact on the greenhouse effect, numerous scientific research institutions have focused on enhancing and upgrading building glass. Various types of glass, capable of reducing solar radiation, have emerged in the market—these include low-E glass [6], colored glaze glass, and heat-reflective glass, among others. These mature products can block the sun’s infrared rays from entering the room and have lower hemispheric emissivity, preventing indoor heat from dispersing outside. However, these mature products continue to block infrared rays from the sun even in winter. Natural heating in winter is particularly important, especially in areas with cold winters and hot summers [7]. Consequently, researchers have shifted their focus towards developing and applying dynamic glass, enabling transparent maintenance structures to provide summer shading and winter natural heating [8,9,10]. Thermochromic (TC) glass, with its temperature-sensitive function, is an example of dynamic glass.

Certain materials can adjust their transmittance in response to changes in the external ambient temperature, and they play a specific regulatory role in controlling visible light and infrared light [11,12,13,14,15]. Based on how the visibility of the material changes with temperature, temperature-sensitive materials can be divided into two categories: thermal scattering and thermochromic. The thermochromic type mainly involves the vanadium dioxide system [16,17]. Vanadium dioxide undergoes a phase transition from a nonmetallic state to a metallic one in a specific temperature range. This transition increases the material’s barrier rate to near-infrared wavelengths, thus reducing solar energy transmittance [18]. However, the practical application of vanadium dioxide is limited due to its transformation temperature of 68 °C and its brown-yellow color [19].

Thermosensitive scattering materials display two states: transparency and atomization. A typical example of these materials is the N-isopropyl propionamide hydrogels, which have been the subject of extensive research and development [20]. These include poly(N-isopropylacrylamide)(PNIPAm) hydrogels crosslinked with N,N-methylene bisacrylamide [21]. When the ambient temperature rises above the phase separation temperature of PNIPAm (34~35 °C), the hydrogel undergoes a noticeable transition from a transparent state to a turbid state. The transmittance contrast (i.e., the difference between the transmittance in the clear and turbid states) exceeds 90%. The phase separation temperature of PNIPAm can be adjusted via copolymerization with acrylamide [22,23]. However, the system’s durability and the material’s temperature sensitivity limitations further constrain the use of this material in smart glass [24]. To reduce the defects of PNIPAm, the researchers began by making it into particles, then mixing and forming smart glass in a gel or water solution [25,26,27]. Additionally, liquid crystals are also used as thermochromic glass with active control [28,29], The researchers prepared a stable liquid crystal film with a smectic A (SmA)-chiral nematic phase (N*) phase transition. Using the oriented film, the system is transparent without electricity, and the product is atomized after heating or electricity. When the temperature exceeds 41.25 °C, the liquid crystal molecules are disordered. The film is atomized and adjusts the visible light. To reduce the transmittance of infrared rays, the authors reduced the infrared rays entering the room by increasing the infrared blocking agent. Hydroxypropyl cellulose is used for studying thermochromic glass and has excellent photothermal regulation ability near 30 °C [30,31]. To obtain better energy-saving and photosensitive effects, researchers began to add infrared-absorbing materials to the hydrogel system, such as cesium tungsten bronze [32]. Some researchers have combined PNIPAm and HPC to make a smart glass with an LCST of 32 °C and a solar adjustment rate of 81% [33]. The triblock polyoxyethylene compound has hydrophilic groups and hydrophobic groups. Sodium dodecyl sulfate (SDS) was used to analyze the effect of active agents on LCST [34]. Subsequently, the researchers made thermosensitive scattering glass with an aqueous solution, but there are many problems in the practical application of an aqueous solution, such as the influence of gravity, and thus the glass cannot change color evenly.

Despite numerous experiments and attempts to develop thermochromic materials suitable for buildings, much of the research remains in the laboratory verification stage. The LCST of the material typically ranges between 30 and 35 °C [35,36,37,38,39]. However, both non-polar and organic temperature-sensitive phase change materials will significantly absorb the heat radiation in the sun. Under the assumption of an air temperature of 25 °C, the glass surface temperature can exceed 40 °C in a short time following solar irradiation. Even in winter, the glass surface temperature will exceed 35 °C or higher in areas with intense solar irradiation. At this time, the glass temperature has exceeded the LCST of the material, and the glass is in a turbid state, meaning it would inhibit the sun’s thermal radiation from entering the room, preventing the room from leveraging solar energy for natural heating in winter. Consequently, the current LCST of the material fails to support the intelligent regulation function of solar energy in high-irradiation regions. In regions with cold winters and hot summers, to optimize the energy-saving effect of smart glass in summer while allowing the sun’s infrared rays to enter the room in winter to reduce artificial heating, the LCST of the thermal dimming material often needs to be set at a temperature above 40 °C or higher. The LCST of triblock polyoxyethylene in an aqueous solution can vary depending on the material’s molecular weight, structure, and HLB value. This variability could be harnessed to create high-performance, temperature-sensitive smart glass that operates at different temperatures.

In this paper, we studied the LCST of triblock polyether with different structures and molecular weights and prepared thermosensitive scattering hydrogel materials by combining them with hydrogels. The thermosensitive scattering (TS) glass was then fabricated by sealing the hydrogel and the glass to form a sandwich structure. This TS glass can measure the overall product LCST, visible light transmission, solar heat gain coefficient (SHGC), and heat transfer coefficient (U). The results show that the LCST of smart glass can be set from 0–70 °C using triblock polymers, sodium chloride, and glycerin. Simultaneously, visible light transmission can be varied from 83% to 8%, and SHGC can be adjusted from 0.682 to 0.31. TS glass still showed excellent stability after 200 hot and cold cycles and 200 h ultraviolet irradiation. The simulation test shows that setting the lower LCST on the top surface results in greater energy savings. However, a higher LCST is required for west window applications for visibility.

## 2. Materials and Methods

### 2.1. Materials

**Figure 1:**
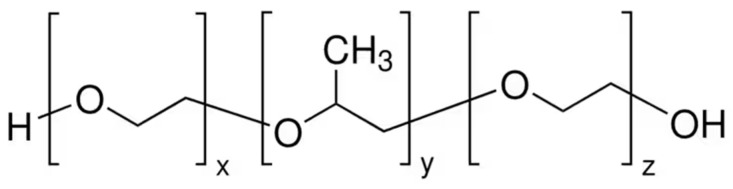
The structure of the triblock polyether.

The temperature-responsive polymer was triblock polyether and was bought from Sigma-Aldrich. Through screening, we selected three kinds of cloud point polyethers for the experiment. According to the technical parameters of the material, E1100 has a cloud point of 37 °C, and the hydrophilic (EO)/hydrophobic (PO) ratio is 10:1. E2000 has a cloud point of 17–21 °C, and EO/PO is 1:9. P2000 has a cloud point of 69 °C, and EO/PO is 1.38:1. The chemical structure of the material is shown in Figure 1 [40].

Acrylamide (AM), glycerol, N,N′-methylene bis (acrylamide) (BIS), ammonium persulfate (APS), sodium alginate (SA), sodium dodecyl sulfate (SDS), and N,N,N′,N′-tetramethylethylenediamine (TEMED) were all bought from Sigma-Aldrich and were without any further purification. Deionized water (18.25 MΩ) was used throughout the experiments.

### 2.2. Cloud Point Test and Thermosensitive Scattering Hydrogel Synthesis

Initially, a 1% aqueous solution of the substance was expertly prepared, and its cloud point was meticulously measured using the water bath method. Following this, the material was utilized to synthesize a thermosensitive scattering hydrogel capable of changing colors.

**Figure 2:**
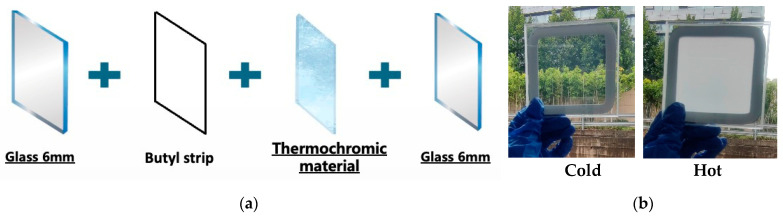
(**a**) The layer structures of the smart glass in this work. (**b**) The clarity changes of the glass in cold and hot environments.

Thermosensitive scattering hydrogel synthesis: triblock polyether, AM, BIS, APS, SA, SDS, and glycerol were dissolved in deionized water in a particular proportion. The ratio of materials depends on the temperature variation. Triblock polyether was 1‰–1% of the total mass of aqueous solution, AM was 15% of the mass of aqueous solution, BIS was 2‰ of the mass of AM, APS was 2% of the mass of aqueous solution, SA was 2% of the mass of aqueous solution, SDS was 50% of the mass of AM triblock polyether, and glycerol was 25% of the mass of deionized water. TEMED was 2.5% of the AM mass. After the material was completely dissolved, the solution temperature was reduced to less than 10 °C, then TEMED was added with agitation, and the solution was kept at −0.085 MPa for 20 min. The material was injected into a double-layer glass with a 2 mm glass cavity (the glass size was 10 cm × 10 cm × 3 mm) (Figure 2a), sealed at the filling port, and placed at 60 °C for curing. The aqueous solution reacts to form a polyacrylamide hydrogel with a sodium alginate structure and a polyacrylamide interpenetrating network in the glass cavity [41,42]. At the same time, the triblock polyether is also uniformly distributed in these network structures. When the hydrogel temperature reaches the set LCST, the triblock polyether is separated from the water phase and finally clusters into small molecular clusters evenly distributed in the hydrogel network. Figure 2b demonstrates that the glass is transparent when its temperature is below the LCST but becomes atomized when it rises above the LCST.

### 2.3. Characterization

Mastersizer 3000 laser diffraction particle size analysis equipment (Malvern Panalytical Ltd., Malvern, UK) was used to test the particle size of the triblock polyether in hot water.

Photothermal performance test: In this experiment, we conducted a photothermal performance test on TS glass using two different types of UV-visible NIR spectrophotometers. The first device was a lambda1050+ (Perkinelmer, Inc., Waltham, MA, USA) with a 150 mm integrating sphere, while the second device was a conventional UV-VIS-NIR spectrophotometer without an integrating sphere (GAG-type glass shading factor product visible light transmittance tester) from Beijing Hongou Chengyun Instrument Equipment Co., Ltd. (Beijing, China). Through these two devices, we were able to test the TS glass’s visible light transmittance (T_vis_), visible light reflectivity (R_vis_), SHGC, and heat transfer coefficient (U). When performing tests on smart glass, it is necessary to use specialized electric heating equipment if the temperature being measured exceeds the LCST (lower critical solution temperature) of the glass. To clarify this concept, let us examine a piece of smart glass with an LCST of 35 °C. We conducted tests on the optical properties of this glass at several temperatures, including 35 °C, 40 °C, 45 °C, 50 °C, and 55 °C.

### 2.4. Weather Resistance Test

The weather resistance test is divided into high- and low-temperature cycle tests and ultraviolet aging tests. The equipment manufacturer is Guangdong Hongzhan Technology Co., Ltd. (Maoming, China). The size of the sample was 100 × 100 mm. The LSCT of the TS glass was 35 °C.

Cold and hot cycle test: The high temperature was 70 °C and kept for 10 min. For low temperature, we set the temperature to 0 °C for 10 min. The temperature heating and cooling rate was 5 °C/min. We cycled it 200 times and tested the T_vis_ of the TS glass every 50 times at 34 °C and SHGC at 45 °C.

Ultraviolet irradiation experiment: Temperature 70 °C, xenon lamp irradiation intensity 550 W/m^2^, wavelength 250–800 nm, continuous irradiation for 200 h, and testing the T_vis_ of the TS glass every 50 h at 34 °C and SHGC at 45 °C.

## 3. Results and Discussion

### 3.1. Cloud Point Test

The LCST of triblock polyether materials should be tested before the preparation of thermosensitive scattering hydrogels. Thermosensitive scattering hydrogels can then be configured according to the LCST of the triblock polyether. The accurate LCST value of the material was tested in a water bath by dissolving it in medium ultrapure water and placing it in a finger bottle. The test results are shown in Table 1.

The main component of the thermosensitive scattering hydrogel contains polyacrylamide, and to reduce the freezing point of the hydrogel, glycerin, salts, and ethylene glycol need to be used. However, adding such materials will reduce the LCST of the final thermosensitive scattering hydrogels. Therefore, we used a different triblock polyether to obtain thermosensitive scattering hydrogels ranging from 0 °C to 70 °C. Through experiments, the triblock polyether was divided, as shown in Table 1.

### 3.2. Testing the Molecular Size of the Triblock Polyether at High Temperatures

**Figure 3:**
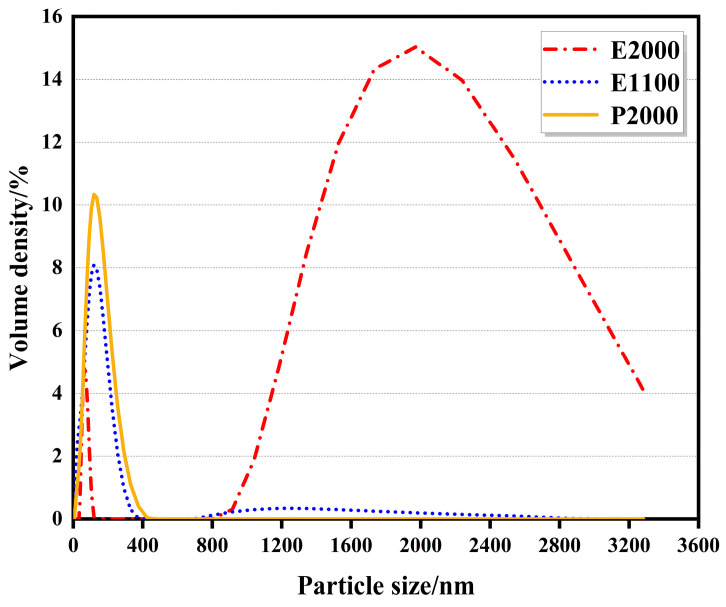
The particle size with the addition of various triblock polyethers.

We used a Mastersizer 3000 to measure the particle size of the triblock polyether after heating in aqueous solution, and the results are shown in Figure 3. According to the findings, E2000 displayed two peaks ranging from 200 to 1900 nm. This material had a cloud point of 27 °C and an HLB value of 3, indicating high solubility at lower temperatures. However, as the temperature of the aqueous solution rose, some of the E2000 separated from the solution. To ensure the accurate testing of the Mastersizer 3000, it is essential to stir the solution, which causes a portion of the precipitated E2000 to disperse into tiny, transparent droplets that can be detected within the 1000–3000 nm range. However, when materials are fully dissolved and heated, the hydrogen bond between the oxygen atoms in the ether bond and the hydrogen atoms in the water breaks, precipitating polyether molecules and forming an oil-in-water emulsion. The test results showed that both E1100 and E2000 particle sizes were similar in hot water solutions. In contrast, P2000 displayed relatively smaller particle sizes in an aqueous solution due to its cloud point at 69 °C. As the hot aqueous solution flowed through the equipment, it lost heat, which caused a drop in temperature. This temperature decrease reduced the agglomeration of P2000 in the aqueous solution, forming relatively more minor molecules. All these particle sizes can be perfectly present in the hydrogel network in polyacrylamide, forming a stable and weather-resistant thermosensitive hydrogel material. The network size of the polyacrylamide hydrogel is about 20 μm [43], which can ensure that the polyether will not settle after being heated. Uniform discolouration of the thermosensitive hydrogel is guaranteed even after multiple cycles. When the triblock polyether particle size is around 200 nm, it has the ability to scatter visible light in the range of sunlight from 380 nm to 780 nm. Due to this, even in the atomized state of TS hydrogel, there will still be some scattered light entering the room. However, this scattered light will block most of the heat and will not have any significant impact on indoor lighting.

### 3.3. Testing the Photothermal Parameters of the Thermosensitive Scattering (TS) Glass

**Figure 4:**
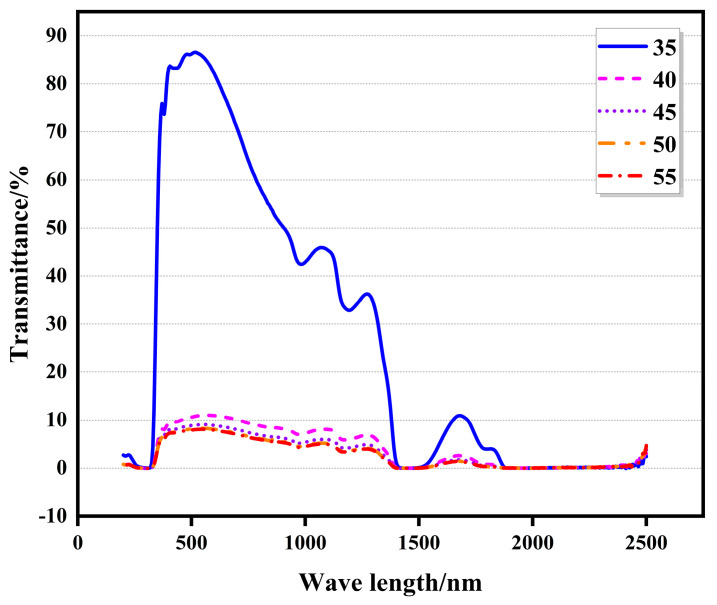
Solar transmission spectra at different temperatures with an integrating sphere (LCST was 35 °C).

**Figure 5:**
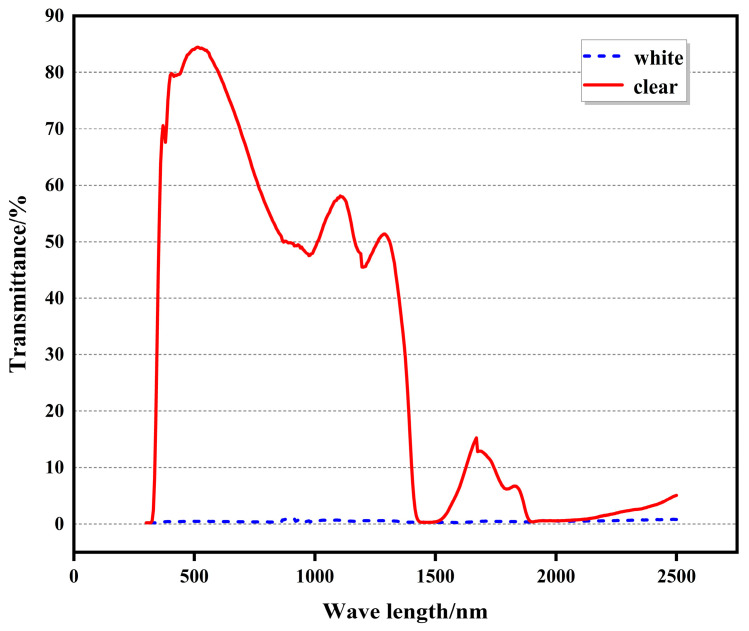
Solar transmission spectra at different clarity states without an integrating sphere (LCST was 35 °C).

In building energy conservation, T_vis_ and SHGC are essential indicators for calculating sunshade performance. In previous studies, the test equipment failed to effectively collect scattered visible and infrared light, resulting in the excessive deviation of the test results. In this study, the photothermal properties of TS glass were tested using a device with an integrating sphere. At the same time, as a comparison, the equipment without integrating the sphere was also tested. As shown in Figure 4, when the TS glass was transparent, it had a high visible light and near-infrared transmittance. When the critical temperature exceeded 35 °C, the transmittance of the thermo-dimming glass decreased rapidly, and when the critical temperature exceeded 10 °C, the transmittance decreased steadily. As the temperature continued to increase, the transmittance of the glass did not decrease much. Figure 5 shows the transmission curves of transparent and atomized states tested without integrating sphere equipment. In the transparent state, the changing trend of the two was similar, and after exceeding the critical temperature, the light transmittance of the TS glass tended towards zero between 300 and 1800 nm. As shown in Figure 3, it can be observed that when the triblock polyether went beyond LCST, the particle size was smaller than the wavelength of the visible and infrared light in the solar spectrum. Consequently, the light in this part of the band w absorbed and scattered. Previous research has shown that the transmittance of many thermally scattered glasses tends towards zero after atomization [27]. This study highlights that devices lacking integrating spheres will miss out on scattered solar radiation, leading to biased test results.

**Figure 6:**
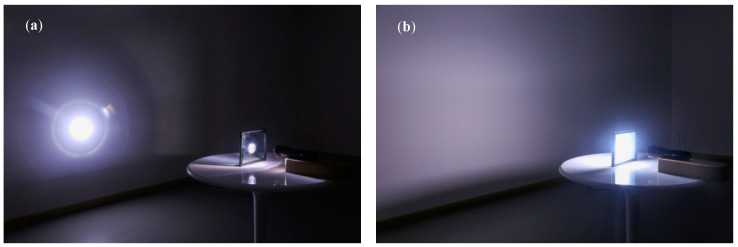
The condition when the same light passed through the TS glass (**a**) before atomization and (**b**) after atomization.

As can be seen from Table 2, TS glass had a significant regulating effect on solar energy. SHGC can have a difference of 0.34. In summer, the shading performance is consistent with the triple silvers glass with the best heat insulation effect on the market, which can significantly reduce the energy consumption of summer air conditioning. In the transparent stage, it is like ordinary glass and can maximize the solar energy into the room to achieve the effect of natural heating. From Table 2, it can be observed that the SHGC of TS glass showed the highest effect from transparent to completely atomized, and the temperature also exceeded 10 °C. However, the wide atomization temperature interval may not be conducive to building energy conservation. By reducing the atomization temperature interval, it is possible to achieve better building energy conservation. T_vis_ of the TS glass was only 7.9% after atomization; under the direct irradiation of the summer sun, the light intensity can reach 100,000 lux. After the visible light passed through the TS glass, it still had 7900 lux visible light entering the room, which was fully able to meet the indoor natural lighting. At the same time, the indoor light was softer because the light was scattered. As shown in Figure 6, after the triblock polyether underwent a phase transition, its particle size was approximately 200 nm. Since the visible light in sunlight was concentrated between 380 and 780 nm and had a visible wavelength greater than the particle size, scattering occurred easily. As a result of multiple light scattering, the light that passed through the TS glass became gentler.

Table 3 shows the photothermal properties of the glass when the TS layer (6G + 2T + 6G) was in different positions. As can be seen from the table, when the TS layer was in the outdoor layer, the SHGC difference between the transparent and atomized states reached 0.3. This structure can maximize the adjustment of solar irradiation in winter and summer so as to reduce the energy consumption of air conditioning by shading in summer and natural heating in winter to reduce the heat load of air conditioning. When the TS layer was in the room, the SHGC from transparent to atomized was only 0.17, and the adjustment ability of sunlight was poor. The TS layer had no significant influence on the heat transfer coefficient, whether it was on the indoor or outdoor side. As can be seen from the table, the TS layer has a strong absorption of solar radiation, so the temperature of the TS layer will increase after atomization. Therefore, the TS layer on the outdoor would be more conducive to energy saving. When the glass surface temperature rises, the heat in the form of far-infrared radiation into the atmosphere, although there will be a heat exchange with the air, can significantly reduce the heat in the room. We can see that TS glass had a good adjustment function for ultraviolet, and the sunlight was able to reduce the ultraviolet light in the light after the TS glass and reduce the photo oxygen aging of ultraviolet light on indoor items.

### 3.4. The Stability of the TS Glass

According to the data in Table 4, the thermosensitive scattering hydrogel displayed exceptional stability when sealed within the glass cavity, even after enduring 200 cycles of extreme hot and cold temperatures and a 200 h ultraviolet irradiation experiment. Despite a slight decrease in photothermal properties of the TS glass following 50 h of ultraviolet exposure, subsequent weather resistance testing indicated that the performance of the TS glass remained steady. This was due to the oxygen-free environment created by the glass cavity, which hindered photothermal aging and thermal oxygen aging. Consequently, as long as the seal is maintained, the TS glass should remain stable in practical applications.

### 3.5. Simulation of the Application of TS Glass to Buildings

**Figure 7:**
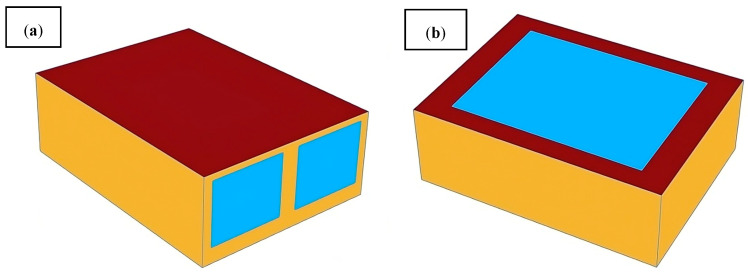
(**a**) The energy saving simulation model of the TS glass from the west window. (**b**) The energy saving simulation model of the TS glass from the skylight (top window).

Low LCST of TS glass has a great energy-saving effect during summer. However, during winter, it can block solar thermal radiation, affecting the glass’s transparency. To evaluate the energy-saving performance of TS (thermosensitive) glass and its visibility at different times, we conducted a photothermal simulation in a room that measures 8 × 6 × 2.7 m. The model was designed with a west-opening window ratio of 0.6 and a skylight-opening window ratio of 0.6, as shown in Figure 7. The simulation tool is based on the Grasshopper platform on Rhino, and the Ladybug Tools plug-in invokes the Energyplus kernel for building performance simulation. The specifications and parameters of the simulated glass are shown in Table 5.

**Figure 8:**
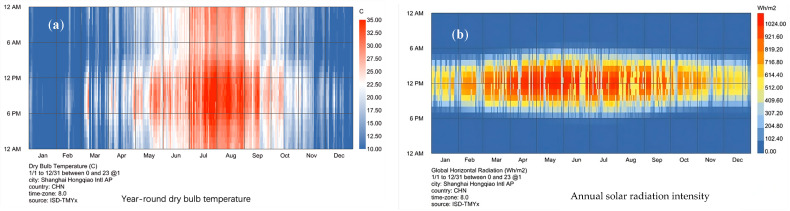
Temperature and irradiation map of Shanghai. (**a**) The year-round temperature in Shanghai; (**b**) the annual solar irradiation in Shanghai.

Shanghai, a region with hot summers and cold winters, was selected as the simulation region. Shanghai also has a tropical monsoon climate with four seasons and full daily life. The overall climate is mild and humid, with short spring and autumn and extended winter and summer. As shown in Figure 8, Shanghai is very hot from July to September, but the radiation intensity in Shanghai is relatively high throughout the year. From March to October, the radiation intensity in Shanghai is very high. Hence, there is the need for summer shading. At the same time, in winter, the temperature is relatively low, but there is still sufficient irradiation intensity. If used, it significantly contributes to energy saving.

**Figure 9:**
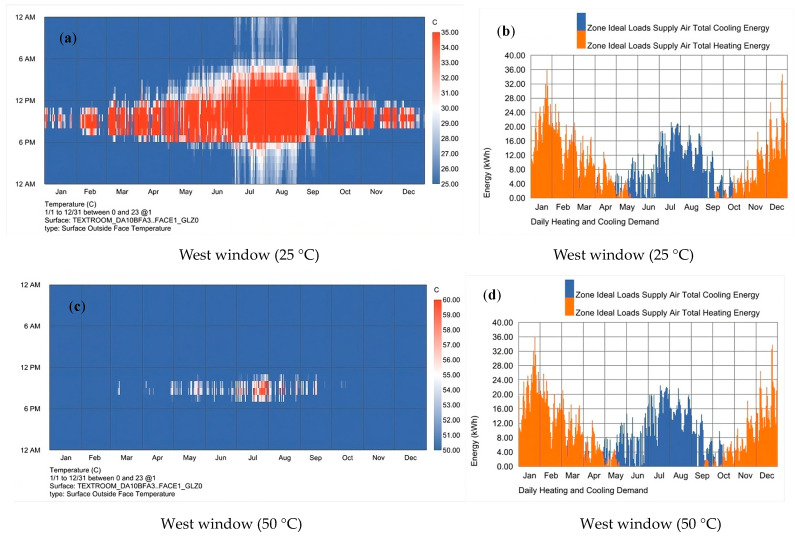
When TS glass was used in the west window. (**a**) The time when the TS glass was transparent and atomized when the temperature was set at 25 °C. The blue part is the glass transparent state, the red part is the glass atomized, and the white area is the glass in a translucent state. (**b**) The annual energy consumption of air conditioners when the glass was at 25 °C. (**c**) The distribution diagram of transparency and atomization at 50 °C. (**d**) The annual energy consumption of air conditioners when the glass was 50 °C.

**Figure 10:**
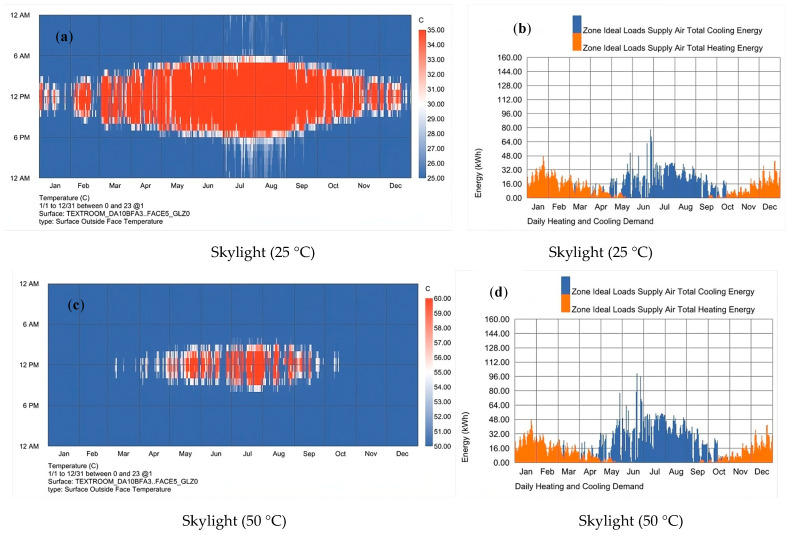
When TS glass was used in the skylight. (**a**) The time when the TS glass was transparent and atomized when the temperature was set at 25 °C. (**b**) The annual energy consumption of air conditioners when the glass at 25 °C. (**c**) The distribution diagram of transparency and atomization at 50 °C. (**d**) The annual energy consumption of air conditioners when the glass at 50 °C.

Figure 9 and Figure 10 show the discoloration time and energy savings when using 25 °C and 50 °C TS glass at the top and west facing sides, respectively. As can be seen from the figure, although 25 °C in the west had a good energy-saving effect in the summer, there was atomization time throughout the year, especially in the hottest summer, from 6 a.m. to 6 p.m., and even in the winter afternoon, the glass was also in the atomization state; at this time, the energy-saving effect range was not as good as 50 °C, as shown in Table 6. In winter, the energy saving effect of TS glass at 50 °C was better than that of 25 °C. Secondly, considering the permeability, in the Shanghai area, the temperature was set at 50 °C, and the glass was in the fog state when it was the hottest in the summer, with the rest of the time it being in the transparent state. Considering the visibility and comfort of the room, a relatively high discoloration temperature should be set on the western façade.

When glass was applied to the skylight, comparing Figure 9b and Figure 10b, the energy consumption at the top of the energy increased significantly. Because the solar irradiation at the top was more robust, more power was needed to improve the indoor thermal comfort when glass was used. By comparing Figure 10b,d, when using 50 °C, the refrigeration energy consumption required in summer increased significantly because with the use of 50 °C TS glass, the glass discoloration time is very short, and too much solar energy would have entered the room before it was completely discolored. Compared with the use of 25 °C, the cooling load was significantly improved. At the same time, due to the thermal insulation performance of glass in winter, there was little difference in energy consumption in winter. Therefore, at the top, the temperature selection needs to choose the temperature with a better energy-saving effect. This study only compared the glass at 25 °C and 50 °C, but the practical application needs to consider both winter and summer. Also, it needs to consider the application scenario so as to achieve the best temperature setting requirements.

From Table 6, it appears that different types of glass have varying energy requirements. It seems that using TS glass is the most effective for energy saving, whether it is summer or winter and whether it is in the west or top position. Interestingly, this is even more effective than the three silver Low-E glasses with the best insulation effect. The energy savings were particularly noticeable in top applications during summer. When the glass is atomized and energy saving, visible light enters the room through scattering, which can reduce the need for artificial lighting.

## 4. Conclusions

In this paper, we developed TS smart glass by encapsulating triblock polyether-PAM hydrogel between two layers of glass. The LCST of the TS glass can be set between 0 and 70 °C. As measured by a spectrophotometer with a 150 mm integrating sphere, the visible light transmittance of the smart glass shifted to 0.78 when transitioning from a transparent to an atomized state. The difference in the SHGC reached 0.37. This effectively controlled the amount of solar heat entering the room in both winter and summer under transparent and atomized states, thereby reducing the load on air conditioning systems in both seasons. Compared to Low-E glass, using 25 °C TS glass at the skylight can save 26% of cooling energy consumption during summer while still contributing to the heat load in winter. Furthermore, by leveraging the scattering state of visible light reflection and transmission, the urban heat island effect can be significantly reduced, and natural indoor lighting can be enhanced. In this study, the photothermal properties of TS glass were tested. However, due to the large amount of water in the material, the hydrogel may freeze in colder regions. Further research is required to reduce the freezing point and ensure the high LCST of the material. Additionally, in practical applications, it is necessary to simulate and engineer the LCST of the glass in a reasonable manner. By incorporating a wide range of temperature points, designers can explore more creative concepts and augment the artistic value of buildings. Our smart glass, designed to respond to temperature changes, offers features like shading and transparency. The result is a safe dynamic glass that requires minimal maintenance and is perfectly suited for use in energy-efficient buildings.

## Figures and Tables

**Table 1:** The LCST of the pure triblock polyether and hydrogels with the addition of the polyether.

Polyether	LCST (°C) (1% Aqueous Solution)	Hydrogel LCST (°C)
E2000	27	≤25
E1100	36	25–50
P2000	73	≥50

**Table 2:** Transmission, reflection, and SHGC of the TS glass at various temperatures.

Test Temperature	T_vis_	R_vis_	SHGC
<35 °C	0.833	0.09	0.68
40 °C	0.109	0.083	0.362
45 °C	0.089	0.107	0.346
50 °C	0.081	0.128	0.337
55 °C	0.079	0.140	0.334

**Table 3:** Optical and thermal parameters of TS glass in different orientations.

Smart Glass		Solar					UV	/	
Status	Structure	T_sol_ *	R_fsol_ *	R_bsol_ *	A_bsol1_ *	A_bsol2_ *	T_uv_ *	SHGC	U(w/m^2^k)
White	(From outside) * 6G + 2T + 6G + 12A + 6G	0.1225	0.1835	0.2039	0.6766	0.0174	0.1476	0.2580	2.7780
	(From outside) 6G + 12A + 6G + 2T + 6G	0.1225	0.2039	0.1835	0.1607	0.5129	0.1476	0.4700	2.7600
Clear	(From outside) 6G + 2T + 6G + 12A + 6G	0.4445	0.0996	0.1264	0.3910	0.0649	0.5728	0.5560	2.7580
	(From outside) 6G + 12A + 6G + 2T + 6G	0.4445	0.1264	0.0996	0.1518	0.2773	0.5473	0.6410	2.7250

* T_sol_: transmittance of solar energy. * R_fsol_: solar reflectance (outdoor measurement). * R_bsol_: reflectance (indoor measurement). * A_bsol1_: direct solar energy absorption ratio (outdoor measurement at the hollow layer). * A_bsol2_: direct solar energy absorption ratio (interior side of the hollow floor). * T_uv_: ultraviolet transmittance. * 6G + 2T + 6G + 12A + 6G: 6G represents 6 mm of ordinary glass, 2T represents 2 mm of TS hydrogel, and 12A means a 12 mm air layer.

**Table 4:** Weather resistance of TC glass with thermal aging mode and UV aging mode.

Aging Mode	Parameter	Test Points (Cycle or h) *				
		0	50	100	150	200
Cold and hot cycle (test points unit: cycle)	T_vis_	83.8%	83.2%	83.1%	83.1%	82.9%
	SHGC	0.35	0.35	0.34	0.34	0.34
Ultraviolet aging (test points unit: h)	T_vis_	83.4%	82.9%	82.5%	82.1%	81.9%
	SHGC	0.35	0.34	0.33	0.33	0.33

* For the cold and hot cycle mode, the unit of test points was ‘times’ (0, 50, 100, …, cycles); for the ultraviolet aging mode, the unit of test points was ‘h’ (0, 50, 100, …, hours).

**Table 5:** The glass parameters used in the simulation in this work.

Types of Glass	U (w/m^2^k)	SHGC (Clear)	SHGC (White)
TS glass (25 °C/50 °C)	1.69	0.31	0.13
Triple silvers glass	1.65	0.21	/
Ordinary glass	2.80	0.67	/

**Table 6:** Energy consumption comparison in winter and summer of different orientations of the glasses.

Orientation	Type of Glass	Sunmmer Cooling Demand (kWh)	Winter Heating Demand (kWh)
West window	Ordinary glass	2705.8	2642.4
	Triple silvers glass	1575.4	2578.3
	TS glass (25 °C)	1484.7	2569.0
	TS glass (50 °C)	1720.8	2534.7
Skylight	Ordinary glass	11135.2	3876.9
	Triple silvers glass	4376.8	3368.9
	TS glass (25 °C)	3204.5	3353.9
	TS glass (50 °C)	5792.6	3307.2

## Data Availability

The data are contained within the article.
